# Transcriptomes from German shepherd dogs reveal differences in immune activity between atopic dermatitis affected and control skin

**DOI:** 10.1007/s00251-020-01169-3

**Published:** 2020-06-18

**Authors:** K. Tengvall, K. Bergvall, M. Olsson, B. Ardesjö-Lundgren, F. H. G. Farias, M. Kierczak, Å. Hedhammar, K. Lindblad-Toh, G. Andersson

**Affiliations:** 1 Science for Life Laboratory, Department of Medical Biochemistry and Microbiology, Uppsala University, Uppsala, Sweden; 2 Department of Clinical Sciences, Swedish University of Agricultural Sciences, Uppsala, Sweden; 3 Division of Rheumatology, Department Medicine, Center for Molecular Medicine, Karolinska Institutet, Solna, Sweden; 4 Department of Psychiatry, Washington University School of Medicine, St. Louis, MO 63110 USA; 5 Department of Cell and Molecular Biology, Infrastructure Sweden, Science for Life Laboratory, Uppsala University, Uppsala, Sweden; 6 Broad Institute of MIT and Harvard, Cambridge, MA USA; 7 Department of Animal Breeding and Genetics, Swedish University of Agricultural Sciences, Uppsala, Sweden

**Keywords:** Canine atopic dermatitis, mRNA sequencing, Differential gene expression, Skin transcriptome

## Abstract

Canine atopic dermatitis (CAD) is an inflammatory and pruritic allergic skin disease with both genetic and environmental risk factors described. We performed mRNA sequencing of non-lesional axillary skin biopsies from nine German shepherd dogs. Obtained RNA sequences were mapped to the dog genome (CanFam3.1) and a high-quality skin transcriptome was generated with 23,510 expressed gene transcripts. Differentially expressed genes (DEGs) were defined by comparing three controls to five treated CAD cases. Using a leave-one-out analysis, we identified seven DEGs: five known to encode proteins with functions related to an activated immune system (*CD209*, *CLEC4G*, *LOC102156842* (*lipopolysaccharide-binding protein-*like), *LOC480601* (*regakine-1*-like), *LOC479668* (*haptoglobin*-like)), one (*OBP*) encoding an odorant-binding protein potentially connected to rhinitis, and the last (*LOC607095*) encoding a novel long non-coding RNA. Furthermore, high mRNA expression of inflammatory genes was found in axillary skin from an untreated mild CAD case compared with healthy skin. In conclusion, we define genes with different expression patterns in CAD case skin helping us understand post-treatment atopic skin. Further studies in larger sample sets are warranted to confirm and to transfer these results into clinical practice.

**Electronic supplementary material:**

The online version of this article (10.1007/s00251-020-01169-3) contains supplementary material, which is available to authorized users.

## Introduction

Canine atopic dermatitis (CAD) is an inflammatory and pruritic allergic skin disease with a strong genetic predisposition and is also influenced by environmental risk factors (Meury et al. 2011; Nodtvedt et al. 2007). Onset of clinical signs is typically between 6 months and 3 years of age in affected dogs. Clinical signs of CAD include eczematous skin, predominantly in the flex and friction areas of the body (Griffin and DeBoer 2001), strikingly similar to symptoms of atopic dermatitis (AD) in humans (Rhodes et al. 1987; Willemse 1988). The immune response during an atopic reaction is primarily a lymphocytic skin infiltration and plasma cell class switching to form immunoglobulin E (IgE) antibodies that recognize otherwise harmless environmental allergens. These IgE antibodies bind to IgE receptors expressed on the cell surface of mast cells and basophils and when IgE are cross-linked by the offending allergen, degranulation and release of inflammatory mediators occur. This leads to vasodilation and activation of further inflammatory responses. The overall prevalence of CAD typically ranges from 3 to 15% (Hillier and Griffin 2001; Williams 2001) and high-risk breeds include boxer, bull terrier, West Highland white terrier, German shepherd dog (GSD), and Labrador retriever (Jaeger et al. 2010; Nodtvedt et al. 2006; Sousa and Marsella 2001; Vilson et al. 2013). Heritability of CAD in Labrador and golden retrievers was estimated to be 0.47 (± 0.17) (Shaw et al. 2004).

The severity of clinical signs, the effect of different treatment protocols, and disease progression differ greatly between different CAD-affected dogs (Olivry et al. 2015). Moreover, many treatments may have adverse side effects (Bloom 2006). Anti-allergic drugs include antihistamines and immune suppressive drugs, e.g., cyclosporine A, glucocorticoids or oclacitinib. Secondary overgrowth or infections with *Malassezia* or *Staphylococcus* spp. are well-recognized flare factors; thus, infection control and prevention is important for successful disease management. Allergen-specific immunotherapy (ASIT) has been developed also for dogs and may, in some patients, be effective in inducing allergen tolerance, hence reducing or even controlling allergic symptoms (reviewed in (DeBoer 2017)). Therapies used for treating dogs with CAD are comparable to treatment of human AD patients (Werfel et al. 2014). The similarities between AD in human and dog both regarding disease presentation (Marsella and Girolomoni 2009), as well as treatment options, make the results obtained from CAD studies potentially useful also for human AD research. The variations between patients in disease progression and severity as well as response to treatment emphasize the need to develop new therapies and personalized treatment strategies in both human and dog AD patients (Cabanillas et al. 2017; Olivry et al. 2015). To further understand the mechanisms underlying CAD, including genetic risk factors, cell types, and molecular pathways, studies of skin in subclinical and active CAD stages are highly warranted.

Differentially expressed genes (DEGs) have previously been reported in a custom-designed 22K gene expression microarray study of both lesional and non-lesional skin from atopic dogs compared to skin from controls (Merryman-Simpson et al. 2008). In that study, 54 DEGs were identified and the most dysregulated gene was *S100A8*, encoding a calcium-binding protein involved in regulating inflammatory responses. Skin biopsies were taken from different parts of the body and various breeds were jointly analyzed. A similar study of human AD identified 217 DEGs of which the most differentially expressed genes encoded proteins involved in inflammatory responses (e.g. *S100A8*, *-A9* and *-A12*, and *CXCL1*) and the skin barrier (e.g., *keratin 16*, and *claudin 8*) (Suarez-Farinas et al. 2015). In a meta-analysis (Ghosh et al. 2015) of five independent human AD microarray studies (Gittler et al. 2012; Guttman-Yassky et al. 2009; Olsson et al. 2006; Plager et al. 2007; Suarez-Farinas et al. 2011), 89 DEGs were identified as consistently dysregulated across all five studies. The defined genes were functionally implicated in immune responses, keratinocyte differentiation/epidermal development, inflammation, and lipid metabolism (Ghosh et al. 2015).

In this study, we collected skin biopsies from the axillary region, which is one of the typically affected body regions in the active disease stage, from CAD-affected and healthy control GSDs. We identified seven significant DEGs comparing treated CAD cases to controls. Post-study design identification of an untreated mild CAD case allowed us to compare untreated atopic versus healthy control skin and indicated inflammatory genes with high expression in the untreated mild CAD dog.

## Methods

### Samples

Skin biopsies were collected from 10 GSDs. The dogs were included in our previous genome-wide association study of CAD in GSDs (Tengvall et al. 2013) and recruited based on their genotypes (cases with risk alleles and healthy controls with control alleles) at the *Plakophilin-2* (*PKP2*)-locus. One biopsy (6 mm in diameter) collected from axillary skin was fixed within 10 min in RNAlater (Ambion Inc., Austin, TX, USA) and stored at 4 °C overnight followed by storage in − 80 °C until RNA extraction.

### CAD and control phenotype characterization

All six cases had been diagnosed with CAD. Clinical diagnoses were established by first ruling out other causes of pruritus such as ectoparasite infestation, staphylococcal pyoderma, and *Malassezia* dermatitis. Hypoallergenic dietary trials (at least 8 weeks followed by a challenge period) were conducted to evaluate the potential contribution of concurrent cutaneous adverse food reactions to the clinical signs. A CAD diagnosis was determined in dogs not adequately controlled on hypoallergenic diet and with positive reactions on intradermal allergy tests or IgE serology tests. The dogs were between 6 and 11 years old at the time of sampling. At the time point of biopsy collections, CAD cases were under treatment with ASIT (administered sub-cutaneous), methylprednisolone/medrol (cortisone), and/or cetirizine (antihistamine) (Table S1).

One dog was originally recruited as a control (control 2), but at the time of sampling, the dermatologist observed mild, non-infectious otitis externa at the examination. The clinical findings warranted an in-depth interview with the owner, which revealed that the dog had experienced summer erythema of inguinal skin and otitis externa at least twice during the last 2 years. These signs are consistent with common clinical signs of CAD (Favrot et al. 2010). An additional axillary skin biopsy from this dog was fixed in 4% PFA, paraffin embedded, and later cut and stained with hematoxylin eosin. The dermatologist observed mild perivascular infiltration of mononuclear cells in superficial dermis. That dog (control 2) was thus post-study design defined as an untreated CAD case with mild skin lesions further referred to as *untreated mild CAD case*. Less than 2 years after sampling, the dog was euthanized due to heart problems and never underwent a complete CAD investigation.

Healthy controls (*n* = 3) were recruited at ages between 9 and 11 years and had no history of either pruritus, repeated otic inflammation, or evidence of skin lesions compatible with CAD, neither prior to nor at the time of sampling. The information was based on owner statement and questionnaire, and later clinical examination at sampling by a boarded dermatologist.

### RNA isolation, library preparation, and sequencing

RNA was isolated using Qiagen RNeasy Mini Kit, Quick-Start Protocol Part 1 and 2 (Jan 2011, www.qiagen.com) with a DNase I digestion step included and the optional step after step 6 in Part 1 excluded (i.e., no new collection tubes were used). Prior to RNA isolation, tissues were homogenized using a bead beater at 4 °C. Beads were washed in 99.9% EtOH for 20 min and then sprayed with RNaseZap RNase Decontamination Solution (Applied Biosystems, Foster City, CA, USA). Poly-A selected/paired-end libraries and sequencing of 100 bp paired-end reads (three lanes) using Illumina HiSeq 2000 were performed at the SNP&SEQ Technology Platform at Science for Life Laboratory, Uppsala University, Sweden. RNA concentration and quality of each sample was assessed at the SNP&SEQ Technology Platform and one sample (case 6 in Table S1) was excluded in the quality control.

### Mapping procedures and quality controls

We used the tool Trimmomatic (v. 0.32) (Bolger et al. 2014) to trim the sequence ends. In total, 93–96% of the input read pairs survived trimming using the Illumina adaptors provided in the TruSeq3-PE.fa with the following settings: 2:30:10 LEADING:3 TRAILING:3 SLIDINGWINDOW:4:15 MINLEN:36. FastQC (v. 0.11.2) (Andrews 2010) was used for evaluating sequence quality and read depth per sample. Bowtie2 (v. 2.2.3) (Langmead and Salzberg 2012) and tophat (v. 2.0.12) (Trapnell et al. 2009) were used to map the reads to the canine genome version CanFam3.1 (Hoeppner et al. 2014) (Ensembl gene annotation released July 2012) and SAMtools view (v. 1.3) (Li et al. 2009) to quality filter the output bam files (−q 15). The functions cuffquant and cuffdiff from the cufflinks (v. 2.2.1) software (Trapnell et al. 2010) were used to quantify the number of aligned transcripts (measured in FPKM, i.e., fragments per kilobase of exon per million reads mapped).

### Definition and visualization of differentially expressed genes

The base 2 log of the fold change for cases FPKM/controls FPKM and a test statistic were calculated using cuffdiff to compute significance of the observed change in FPKM (GitHub commit 15d2c6b). We used the default (0.05) setting for significant FDR in cuffdiff. Analyses for defining DEGs were performed by comparing all five CAD cases with three healthy controls and also by using a *leave-one-out* approach defining DEGs in common between eight comparisons of cases and controls, where one dog was subsequently omitted to avoid effects from individual variation in gene expression. We also performed a comparison between the untreated mild CAD case with the healthy controls. As quality control, we excluded DEGs with < 1.5 log2 fold change and DEGs with < 10 FPKM in more than 50% of the samples. In the comparison between the untreated CAD case compared to controls, we applied an additional quality control by subsequently excluding DEGs with less than double/half FPKM difference between the untreated case and any of the other individual FPKM (both controls and treated cases). R package CummeRbund (v. 2.14.0) (Goff L 2013) was used to evaluate and visualize the expression results returned by cuffdiff. R package gplots (v.3.0.3) (Warnes R G 2020) and Adobe Illustrator 2019 (v. 23.0.6) was used for creating final figures.

## Results

### Total mRNA expression in dog skin

In total, expression of 23,510 gene transcripts (including 6440 *LOC* genes), 48,265 isoforms, 36,295 transcription start sites (TSS), and 23,509 promoters were detected in the dog skin samples. All samples remaining after quality control at the sequencing platform passed the threshold of sequence quality (mean PHRED score > 31), and aligned reads per sample ranged from 36.8 to 45.6 million. Control samples showed higher within-group variation (coefficient of variation, CV^2^) in comparison to cases (Fig. S1A). Multi-dimensional scaling (MDS) and principal component analyses (PCA) visualizing the overall gene expression per individual showed no grouping based on cases and control status (Fig. S1B-C) and FPKM was similar across individual samples (Fig. S1D).

### Differential gene expression in treated CAD cases versus controls

**Fig. 1:**
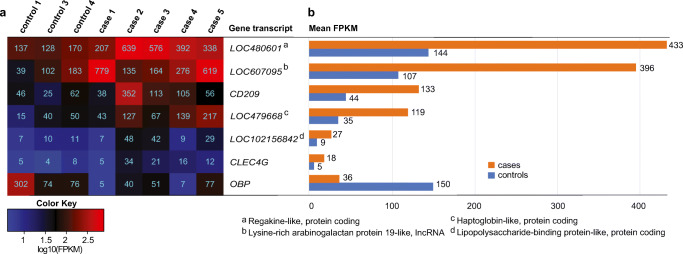
Differentially expressed genes in CAD cases compared to controls. Expression levels for seven DEGs from the *leave-one-out* analysis of CAD cases compared to controls are presented as FPKM (fragments per kilobase of exon per million reads mapped) in a heatmap, color coded based on log10-transformed values and the actual values presented as numbers in each square (**a**). Mean FPKM in the case and control groups are presented as staple bars (**b**). Description of *LOC* gene transcripts is referred to with superscripts a–d. *OBP* showed higher expression in cases whereas the others presented with lower expression levels in cases

In the comparison between five CAD cases and three controls, 135 DEGs (Table S2) were identified and no expression differences between CAD cases and controls were detected for the *PKP2* gene, previously reported associated with CAD (Tengvall et al. 2013). Seven DEGs were defined after two quality controls. The first quality control resulted in eight DEGs (> 1.5-fold change and with FPKM > 10 in at least 50% of the samples). The second quality control was a leave-one-out analysis resulting in 10 DEGs. The seven DEGs overlapping between both quality controls were *CD209*, *CLEC4G*, *LOC102156842* (*lipopolysaccharide-binding protein like*), *LOC480601* (*regakine-1-like*), *LOC479668* (*haptoglobin-like*), *LOC607095* (*lysine-rich arabinogalactan protein 19-like*), and *OBP* (Fig. 1). *OBP* showed lower gene expression levels whilst the other genes showed higher gene expression levels in cases compared to controls.

### Differential gene expression in untreated mild CAD case versus controls

**Fig. 2:**
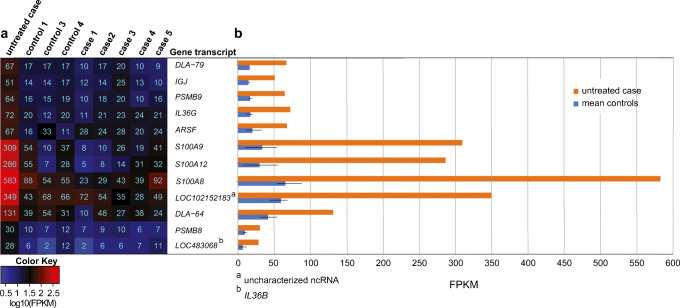
Differentially expressed genes in an untreated mild case compared to controls. Expression levels for 12 DEGs are presented as FPKM (fragments per kilobase of exon per million reads mapped) in a heatmap, color coded based on log10-transformed values and the actual values presented as numbers in each square (**a**). FPKM in the untreated mild case and mean FPKM in controls are presented as staple bars with the range from lowest to highest FPKM in the controls visualized as black strokes (**b**). Description of *LOC* gene transcripts is referred to with superscript a, b

One dog (control 2) was recruited as a control but later identified as an untreated mild CAD case post-study design (see “Methods” and Table S1). We compared the mRNA expression levels of this single dog to three healthy controls and identified 45 DEGs after exclusion of DEGs with log2 fold change < 1.5 and with < 10 FPKM in > 50% of samples (Table S3). Extracting DEGs with at least half/double FPKM in the untreated case compared to the other individuals (both controls and treated cases) resulted in 12 DEGs, all with higher expression in the untreated case (Fig. 2). These were *S100A8*, *S100A9*, *S100A12*, *LOC102152183* (uncharacterized ncRNA), *IL36G*, *LOC483068* (*IL36B*), *DLA-79*, *PSMB9*, *IGJ*, *ARSF*, *DLA-64*, and *PSMB.*

## Discussion

### Dog skin transcriptome

The primary aim of this study was to analyze differential gene expression changes specific for CAD skin. By using high-quality RNA and low density of samples (*n* = 3) per lane in sequencing, thus yielding a high number (37–46 M) of reads per sample, a high-quality canine skin transcriptome was obtained. Gene expression data from skin of nine individual dogs of high quality and coverage can now be used to update both the current dog genome annotation version (Hoeppner et al. 2014), which has been lacking high-quality data from the canine skin transcriptome, and the recently developed database for genetic and epigenetic data from dog tissue samples (Megquier et al. 2019).

### Unique study design

In contrast to previous atopic skin gene expression studies, we collected biopsies from the same skin location (axillary region) in both cases and controls from a single dog breed, to limit changes in gene expression due to body location and inter-breed variation. The treatment protocols for the five CAD cases were comparable including allergen-specific immune therapy (ASIT) aimed at inducing allergen tolerance, systemic corticosteroids, and/or antihistamines. At the time of sampling, CAD cases were clinically in remission as a result of the treatment protocol, with no active skin lesions and without pruritus or just mild pruritus; thus, the skin from these treated atopic cases was visually similar to control dog skins. Treatment protocols included methylprednisolone used in the dose range of 0.2–0.35 mg/kg every other day, and it is well known that corticosteroids have immunosuppressive properties (Bonagura 1995). Moreover, cetirizine was used in the dose 1 mg/kg BID and antihistamine blocks histamine receptors and stabilizes mast cells (Ekstrand et al. 2018). The overall gene expression profiles provided from this study showed that cases had a lower within-group variation compared to controls, which may be explained by the similar disease state in cases (i.e., CAD cases were under similar treatment protocols).

### Activated immune response in subclinical atopic skin

Interestingly, non-lesional (i.e., clinically normal) skin from CAD-affected dogs is not identical to skin from healthy dogs. Non-lesional CAD skin has previously been characterized by microscopic inflammation and presence of pro-inflammatory cytokines, similar to lesional atopic skin (Nuttall et al. 2002a; Nuttall et al. 2002b; Olivry et al. 1999; Olivry et al. 1997). T-cells are known to play a crucial role in both the acute and chronic phase of AD/CAD where acute inflammatory response in AD is characterized by Th2-type cytokines (Bieber 2010). Among the seven DEGs identified in treated CAD cases versus controls, were genes encoding proteins that could account for immunological alertness and sensitivity to relapse. *CD209*, *CLEC4G*, and *LOC102156842* (*lipopolysaccharide-binding protein like*) encode proteins involved in early immediate defense against pathogens, and *LOC480601* (*regakine-1-like*) and *LOC479668* (*haptoglobin-like*) encode proteins involved in monitoring inflammatory responses in the skin. *LOC607095* encodes a novel long non-coding RNA, a potential new marker for skin inflammation, and *OBP* encodes an odorant-binding protein potentially related to the AD-connected symptom rhinitis.

*CD209* (alias: *CLEC4L*) encodes a pattern recognition receptor, expressed on macrophages and dendritic cells, and binds to mannose-type carbohydrates commonly found on pathogens. The increased expression level of *CD209* in CAD cases versus healthy controls may reflect an activated interaction between antigen-presenting cells and T-cells in CAD skin tissue. Previously, a reduction of CD209^+^ dendritic cells in human skin from atopic eczema patients was found associated with clinical improvement (Hassan et al. 2007). *CLEC4G* is positioned in the vicinity (~ 45 kb) of *CD209* in the canine genome, and both genes encode proteins with similar functions as detectors of antigens. An upregulated expression of *CLEC4G* was also detected in a previous study of gene expression in acute lesional AD skin from dogs (Plager et al. 2012). *LOC102156842* encodes a lipopolysaccharide-binding (LPB)-like protein. LPBs are acute-phase proteins that recognize lipopolysaccharide (LPS) on bacteria and provide an early inflammatory response essential for defense against invading microorganisms. High LPB concentrations in human serum were shown to reduce LPS activity (Zweigner et al. 2001). *LOC480601* encodes a regakine-1-like protein. Regakine-1 is a CC chemokine that synergizes with IL-8 (CXC chemokine ligand 8) to chemoattract neutrophils and potentiate the inflammatory response in blood circulation (Gouwy et al. 2002). Higher gene expression levels of *CXCL8* were detected in purified epidermal cells from AD patients compared to normal skin (Kamsteeg et al. 2010). *LOC479668* encodes a Haptoglobin-like protein. Haptoglobins are acute-phase proteins shown to prevent epidermal Langerhans cells from functionally maturing in the skin, which may be important for preventing T cell–dependent inflammatory skin disease (Xie et al. 2000). Patients with skin diseases, e.g., psoriasis, had a significantly increased haptoglobin mRNA expression in epidermal keratinocytes compared to controls, and it was suggested that keratinocyte-derived haptoglobin may contribute to the downregulation of inflammatory responses in the skin (Li et al. 2005). Different haptoglobin genotypes have been reported with higher risk of disease in humans (Andersen et al. 2017) including allergic contact dermatitis (Beckman et al. 1981), bronchial asthma (Frohlander and Stjernberg 1989), and rhinitis (Piessens et al. 1984). *LOC607095* corresponds to the Ensembl gene id: ENSCAFG00000041925 and is described as a lysine-rich arabinogalactan protein 19-like (Ensembl release 100, April 2020). The gene encodes nine splice variants of lncRNA where one (ENSCAFG00000079341.1) matches the position to the LOC607095 transcript (chr27:483,001-486,492, Table S2). Recently, there has been an increased focus on how long non-coding RNA species function as critical regulators of immune cell development, differentiation, and effector function, and also how they may be targeted therapeutically. Dysregulated lncRNA has been suggested in both cancer, autoimmunity, and asthma (reviewed in (Guidi et al. 2020)). *OBP* presented with lower expression levels in CAD cases compared to controls. OBP encodes odorant-binding proteins, which are small and abundant extracellular proteins detected in many species and specifically in the human olfactory mucus. They are participating in odor detection by carrying, deactivation, and/or selecting odorant molecules (Briand et al. 2002). Rhinitis, i.e., inflammation in the nasal passages, affects > 1/3 of human AD patients (Kapoor et al. 2008). While not as common in dogs, this clinical feature still affected around 7% of CAD-affected dogs (Favrot et al. 2010) and showed breed variations with the highest proportions reported in CAD-affected GSDs (8.8%) and West Highland white terriers (10.9%) but none of the Dalmatians (Wilhem et al. 2011). The altered expression of *OBP* seen in CAD cases compared to controls could potentially be a secondary effect from rhinitis.

Interestingly, the leave-one-out method defined 10 DEGs, out of which seven were included among the eight DEGs defined after the other quality control excluding DEGs with < 1.5-fold change and FPKM below 10 in > 50% of the individuals. This approach confirmed the validity of the two cutoff methods used to define DEGs.

### Differentially expressed immune genes indicated in untreated mild CAD skin

The majority of DEGs with higher expression in the untreated mild CAD case compared to controls have known functions in an activated immune response and inflammation. *S100A8*, *S100A9*, and *S100A12* encode proteins that are well-known markers of acute inflammation and previously implicated in several other inflammatory and autoimmune diseases including systemic lupus erythematosus, rheumatoid arthritis, and atherosclerosis (Austermann et al. 2018; Oesterle and Bowman 2015). IL-36 cytokines, including IL-36B and IL-36G, actively propagate skin inflammation by activating keratinocytes, antigen-presenting cells, and indirectly T-cells (Foster et al. 2014). *IGJ* encodes the immunoglobulin-joining chain of multimeric IgA and IgM, *DLA-79* and *DLA-64* are part of MHC class I, and *PSMB8* and *PSMB9* are located in the MHC class II region and encode members of the immunoproteasome that are critical for processing of MHC class I peptides (www.ncbi.nlm.nih.gov, Gene database, May 2020). *ARSF* belong to the family of sulfatases and has been suggested as a new marker for psoriasis in a mRNA-seq analysis of skin in humans (Li et al. 2020). *LOC102152183* is an uncharacterized ncRNA and has no previously known functions but could potentially be a novel marker for skin inflammation. Due to the inclusion of only one untreated CAD case in this comparison, no certain conclusions can be drawn, but these suggestive DEGs encoding proteins with striking immunological functions and one novel ncRNA warrant further studies to confirm these findings.

### No expression differences for CAD-associated Plakophilin-2 gene

The dogs in this study were recruited based on their genotypes at the *PKP2*-locus as this was previously found associated with CAD in GSDs (Tengvall et al. 2013; Tengvall et al. 2016). However, we detected no difference in *PKP2* gene expression in the skin between the CAD cases carrying risk alleles compared to the controls with the control genotype. Two additional biopsies from axillary skin and three biopsies from the back region of each dog were included at sampling and were fixed for protein expression studies (Ardesjo-Lundgren et al. 2017), which included a few more dogs compared to the present study. In that study, T-cells and dendritic cell infiltration were identified in canine axillary skin and high PKP2 protein expression was reported in keratinocytes, T-cells, and dendritic cells, but with no differences between CAD cases and controls. The majority of the CAD cases had been under long-term systemic corticosteroid treatment (> 1 year) at the time of sampling (Ardesjo-Lundgren et al. 2017), and corticosteroids are known to inhibit activation of especially lymphocytes but also dendritic cells (Ashwell et al. 2000; Ashworth et al. 1988; Gillis et al. 1979; Leung and Bloom 2003). Since a skin biopsy consists of many cell types, a difference in *PKP2* gene expression in specific cell types such as T-cells or dendritic cells may be undetectable. However, no difference in PKP2 protein expression intensity in dendritic cells was detected between CAD cases and controls (Ardesjo-Lundgren et al. 2017). Nevertheless, we cannot rule out that there is an altered PKP2 expression in CAD but the right target tissue, cell type, and/or disease state need to be defined. Overall, DEGs identified in this study may be caused by genetic alterations associated with CAD; however, in a small sample set like this, differential gene expression likely reflect the actual skin status being either untreated mild CAD, post-treatment subclinical CAD, or healthy tissue.

### Future objectives

The majority of the top DEGs detected from the current analyses were highly supported by previous studies in both dog and human. We here define differential gene expression in subclinical treated CAD skin. The DEGs identified here encode proteins that may represent suitable target molecules. Further studies to detect additional DEGs and confirm the currently defined DEGs are warranted and would preferably involve skin samples from more controls and cases at different disease stages, including active lesional disease as well as treated subclinical CAD cases. Identifying specific therapeutic target molecules and biomarkers may improve the development of future diagnostic tools and therapies to become personalized to increase efficiency and reduce side effects.

## Conclusion

We here generate a high-quality canine skin transcriptome from healthy controls and CAD-affected dogs representing a subclinical phenotype as an effect of treatment. We provide insights into immunological mechanisms that might account for the relapsing nature of atopic disease. Our results emphasize the striking similarities between canine and human AD also at the level of perturbed gene expression profiles in affected compared to healthy skin. These results will hopefully contribute to the foundation of future treatment strategies.

## Electronic supplementary material

ESM 1 (DOCX 1592 kb)

ESM 2 (XLSX 33 kb)

## Data Availability

The raw files (.fastq.gz) are uploaded on ENA (European nucleotide archive: https://www.ebi.ac.uk/ena/) with project accession number: PRJEB38104 (ERP121487).

## Code availability

All software programs and versions used for mapping of reads and analyses are specified in the materials and methods section.
