# Supplementary material for: Transcriptomes from German shepherd dogs reveal differences in immune activity between atopic dermatitis affected and control skin
Source: Immunogenetics. 2020 Jun 18;72(5):315–23. doi: 10.1007/s00251-020-01169-3 (PMC7320941; doi:10.1007/s00251-020-01169-3)
Supplement: Supplementary file 1 — (DOCX 1592 kb) [file 251_2020_1169_MOESM1_ESM.docx]

# Supplemental information

## Supplemental figure

**Fig. S1 Quality estimates of overall FPKM in case and control samples** Control samples showed higher within-group variation (coefficient of variation, CV^2^) in comparison to CAD cases (control 2 *i.e.* the untreated CAD case excluded) (**a**). Multi-dimensional scaling (MDS) in (**b**) and principal component analyses (PCA) in (**c**) of the overall gene expression per individual showed no grouping based on case and control status and log(FPKM) was similar across individual samples (**d**).

**Supplemental tables**

**Table S1-S3.**
